# Gastrointestinal Hemorrhage Severity Triage: Locally Derived Score May Outperform Existing Scoring Systems

**DOI:** 10.14740/gr652w

**Published:** 2015-04-03

**Authors:** Rangson Chaikitamnuaychok, Jayanton Patumanond

**Affiliations:** a Department of General Surgery, Kamphaeng Phet Hospital, Kamphaeng Phet 62000, Thailand; b Department of Clinical Epidemiology and Clinical Statistics, Faculty of Medicine; Center of Excellence in Applied Epidemiology, Thammasat University, Pathum Thani 12120, Thailand

**Keywords:** Clinical prediction rules, Scoring system, Screening, Upper gastrointestinal bleeding, Upper gastrointestinal hemorrhage, Validation

## Abstract

**Background:**

Scoring tools to predict need for intervention, re-bleeding and mortality of upper gastrointestinal hemorrhage (UGIH) have been developed. It is inconclusive whether these tools are also appropriate for UGIH severity and/or urgency triage. The objective of the study was to compare the performances of the Blatchford score, the Rockall score, and the UGIH score on UGIH severity triage.

**Methods:**

Retrospective 3-year data of UGIH patients (2009 - 2011) were collected. Patients were assigned to each of the three scoring systems based on their clinical characteristics required for the scoring systems. The score ranges of each scoring system were transformed into the same scale from 0 to 100. The score performances were compared by diagnostic indices, graphically presented with area under receiver operating curve (AuROC), discrimination curves, and statistically tested with Chi-squared tests.

**Results:**

When focusing on the diagnostic indices, the local UGIH had similar sensitivity to, but better specificity than the Blatchford score in detecting mild UGIH. The sensitivity was better than and the specificity was less than the Blatchford score in detecting severe UGIH. The local UGIH score was better than the pre-endoscopic Rockall in almost all diagnostic indices. Focusing overall performances, the local UGIH score classified patients non-significantly better than the Blatchford: 89.3% vs. 87.9% for mild (P = 0.243), 87.2% vs. 85.0% for severe (P = 0.092), but significantly classified better than the pre-endoscopic Rockall score: 89.3% vs. 76.4% for mild (P < 0.001), and 87.2% vs. 81.2% for severe (P < 0.001). When exploring the discrimination curves, the Blatchford score classified more patients into the mild categories, and less into the severe categories than the local UGIH score. In contrast, the pre-endoscopic Rockall score classified less patients into the mild, but more into the severe than the local UGIH score.

**Conclusion:**

Triaging UGIH patients into three severity levels in order to decide or set for endoscopy should apply the scoring system specifically developed for that purpose. Adopting other scores developed for other purposes may result in under- and/or over-estimations. The local UGIH score classified patients into three severity levels to help indicate endoscopy more efficiently than the Blatchford score and the pre-endoscopic Rockall score which was developed for different purposes.

## Introduction

Upper gastrointestinal tract hemorrhage (UGIH) is one of the most common causes of hospital admission. The severity of UGIH varies from mild coffee ground vomiting to massive bleeding (exsanguination) [1]. UGIH severity may be classified by clinical and/or endoscopic criteria [2-11]. In general, “low risk” means re-bleeding < 5% and/or mortality < 1% [12]. Limitations of classifying UGIH severity included debates on, whether it is an absolute indication for endoscopy, whether to manage patients as in-patient department (IPD) or out-patient department (OPD) cases, should early endoscopy be advised in every institute, and were classifications based on various techniques and systems disseminated to the other institutes [12]. It is well accepted that emergent endoscopy should be set in patients with unstable hemodynamics. These may include orthostatic hypotension, tachycardia, shock, and/or signs of continued bleeding [13]. However, emergent endoscopy is not normally set on weekends in most hospitals [1]. Non-emergent or non-urgent cases would therefore be postponed to the next available weekdays, or possibly longer for cases requiring semi-elective procedures [1].

Prior to the time of endoscopy, prediction for UGIH severity was important in prognostication for need of hospitalization, and/or need for close monitoring in intensive care units when patients had high risks for re-bleeding [13]. While clinical parameters alone may be sufficient to predict the possibility of management as OPD cases in patients with low risks for re-bleeding [13].

Many researchers had developed and published scoring systems with an objective to discriminate patients with low risks from those with high risks [3, 5, 6, 8, 14]. The most mentioned and referenced works were the Blatchford score, to predict need for intervention of UGIH [15], and the Rockall score to predict re-bleeding and mortality [5].

We previously developed a local UGIH score (from the data of patients between 2009 and 2010) [16, 17], to triage patients into three severity levels: mild, moderate, and severe [16], based on clinical and laboratory variables. The triaging focused on providing clinical guideline for treatments of UGIH and when to do endoscopy: non-urgent, urgent, and emergent [16]. The developed local UGIH score was internally validated to another independent patient of the following year (from data of patients in 2011) [18]. The local UGIH score performed similarly (both calibration and discrimination) in the development dataset and the validation dataset, and was clinically acceptable [18].

In the present study, we focused on comparing our local UGIH score to the Blatchford score and the pre-endoscopic Rockall score, on their abilities to predict the severity, or urgency of UGIH prior to endoscopy.

## Patients and Methods

### Patients

The UGIH patients were registered in KamphaengPhet Hospital, a university-affiliated tertiary hospital in the northern region of Thailand. We retrieved medical folders of patients who presented to the emergency department with upper gastrointestinal bleeding, between 2009, 2010 and 2011 [16-18]. The ICD-10 keywords for hospital database search were K920-hematemesis, K921-melena and K922-gastrointestinal hemorrhage unspecified.

### Definitions of UGIH severity: an outcome of interest

We used the following criteria to define UGIH severity. 1) Severe: patients who required surgical interventions to stop bleeding, patients in states of grade 3 and 4 shock [19], and patients who did not survive. 2) Moderate: patients who required endoscopy to stop bleeding (endotherapy), patients with re-bleeding, patients in states of grade 1 and 2 shock [19], and patients who required blood transfusion. 3) Mild: patients with no signs of shock, patients who required endoscopy without hemostasis, and patients who did not required any blood transfusions.

### The three scoring systems

We applied the three scoring systems to the same set of patients to compare their performances. 1) The Blatchford Score, which included clinical and laboratory variables (Table 1) [15]. 2) The pre-endoscopic Rockall criteria, which included only clinical variables (Table 2) [5]. 3) The local UGIH score criteria, which included simple clinical and laboratory variables (Table 3) [18].

**Table 1:** Scoring Scheme for the Blatchford Score

Item indicators		Categories	Score
BUN (mg/dL)		< 18.2	0
		18.2 - 22.3	2
		22.4 - 27.9	3
		28 - 69.9	4
		≥ 70	6
Hemoglobin (g/dL)	Male	> 13	0
		12.0 - 12.9	1
		10.0 - 11.9	3
		< 10.0	6
	Female	> 12	0
		10.0 - 11.9	1
		< 10.0	6
Systolic blood pressure (mm Hg)		≥ 110	0
		100 - 109	1
		90 - 99	2
		< 90	3
Pulse rate (/min)		< 100	0
		≥ 100	1
Melena		Yes	1
		No	0
Syncope		Yes	2
		No	0
Hepatic diseases		Yes	2
		No	0
Cardiac failure		Yes	2
		No	0

**Table 2:** Scoring Scheme for the Pre-Endoscopic Rockall Score

Item indicators	Categories	Criteria	Score
Age (years)	< 60		0
	60 - 79		1
	≥ 80		2
Shock	No shock	SBP > 100 mm Hg	0
		Pulse < 100/min	
	Tachycardia	SBP > 100 mm Hg	1
		Pulse > 100/min	
	Hypotension	SBP < 100 mm Hg	2
Comorbidity	No major comorbidity		0
	Cardiac failure		2
	Ischemic heart disease		
	Any major comorbidity		
	Renal or liver failure		3
	Disseminated malignancy		

**Table 3:** Scoring Scheme for the Local UGIH Score

Item indicators	Categories	Score
Age (years)	≥ 60	1
	< 60	0
Pulse (/min)	≥ 100	1
	< 100	0
Systolic blood pressure (mm Hg)	< 100	10.5
	≥ 100	0
Hemoglobin (g/dL)	< 10	6
	≥ 10	0
BUN (mg/dL)	≥ 35	2
	< 35	0
Cirrhosis	Yes	2
	No	0
Hepatic failure	Yes	4.5
	No	0

### Data analysis

Characteristics of patients with mild, moderate, and severe UGIH were described and compared with parametric or non-parametric tests for trend as appropriate. A UGIH severity was predicted based on the three mentioned systems. The minimum and maximum score points of the three systems were converted to the same minimum and maximum scores (0 and 100). Backward calculation was done to obtain standardized score (0 - 100) for each of the three systems. The performances of the scores in classifying patients into three severity scores were presented with classical diagnostic indices: sensitivity, specificity, predictive value of positive, predictive value of negative, and % correctly classified, and tested with exact probability. The prediction of the scores was graphically presented with discrimination curves and tested with an area under the receiver operating curve (AuROC).

## Results

Patients in the three severity groups differed according to age, presence of melena, syncope, systolic blood pressure, hemoglobin, blood urea nitrogen (BUN), presence of cirrhosis, and renal failure.

When the three systems were used to assign scores to each patient in the three severity categories, the mean (± SD) Blatchford scores were 5.0 ± 3.8 in mild group, 10.3 ± 2.7 in moderate group, and 12.0 ± 2.7 in severe group (P < 0.001). The mean (±SD) pre-endoscopic Rockall scores were 0.7 ± 0.8, 1.2 ± 1.2, and 2.7 ± 1.3 (P < 0.001), and the mean (±SD) local UGIH severity scores were 3.3 ± 4.1, 9.0 ± 4.2, and 16.7 ± 4.0 (P < 0.001) (Table 4).

**Table 4:** Characteristics of Patients With Upper Gastrointestinal Hemorrhage and Score Distributions

Characteristics	UGIH severity			P-value
	Mild (mean ± SD)	Moderate (mean ± SD)	Severe (mean ± SD)	
Demographic and presentations				
Age (years)	53.0 ± 18.3	61.2 ± 14.5	58.7 ± 14.6	< 0.001
Hematemesis, n (%)	164 (49.4)	360 (42.5)	113 (49.6)	0.738
Coffee ground vomiting, n (%)	85 (25.6)	140 (16.5)	47 (20.6)	0.054
Hematochezia, n (%)	22 (6.6)	53 (6.3)	23 (10.1)	0.168
Melena, n (%)	141 (42.5)	531 (62.7)	129 (56.6)	< 0.001
Syncope, n (%)	39 (11.8)	172 (20.3)	54 (23.7)	< 0.001
Vital signs and biochemicals				
Pulse (/min)	90.2 ± 16.3	91.5 ± 15.7	93.2 ± 16.6	0.094
Systolic blood pressure (mm Hg)	129.6 ± 20.3	123.8 ± 19.4	90.7 ± 15.8	< 0.001
Hemoglobin (g/dL)	11.7 ± 2.4	6.9 ± 2.1	7.3 ± 2.6	< 0.001
BUN (mg/dL)	23.4 ± 16.4	37.0 ± 22.1	38.5 ± 22.7	< 0.001
Co-morbidity				
Cirrhosis, n (%)	20 (6.0)	123 (14.5)	53 (23.2)	< 0.001
Hepatic failure, n (%)	2 (0.6)	8 (0.9)	6 (2.6)	0.038
Cardiac failure, n (%)	1 (0.3)	6 (0.7)	5 (2.2)	0.024
Renal failure, n (%)	5 (1.5)	82 (9.7)	32 (14.0)	< 0.001
Clinical outcomes				
Re-bleeding, n (%)	7 (2.1)	44 (5.19)	27 (11.8)	< 0.001
Dead, n (%)	0 (0)	2 (0.24)	38 (16.7)	< 0.001
Score types				
The Blatchford score	5.0 ± 3.8	10.3 ± 2.7	12.0 ± 2.7	< 0.001
The pre-endoscopic Rockall score	0.7 ± 0.8	1.2 ± 1.2	2.7 ± 1.3	< 0.001
The local UGIH score	3.3 ± 4.1	9.0 ± 4.2	16.7 ± 4.0	< 0.001

The local UGIH score discriminated mild UGIH patients from the remaining patients correctly in 86.8%, similar to the Blatchford score (87.3%) (P = 0.693), but significantly better than the pre-endoscopic Rockall (70.0%) (P < 0.001).

The local UGIH score also discriminated severe UGIH patients from the other two categories in 90.5%, better than the Blatchford score (74.6%) (P < 0.001), and better than the pre-endoscopic Rockall score (86.1%) (P < 0.001).

When focusing on the diagnostic indices, the local UGIH had similar sensitivity to, but better specificity than the Blatchford score in detecting mild UGIH. The sensitivity was better than and the specificity was less than the Blatchford score in detecting severe UIGH. However, the local UGIH score was better than the pre-endoscopic Rockall in almost all diagnostic indices.

Focusing overall performances, the local UGIH score classified patients non-significantly better than the Blatchford: 89.3% vs. 87.9% for mild (P = 0.243), 87.2% vs. 85.0% for severe (P = 0.092), but significantly classified better than the pre-endoscopic Rockall score: 89.3% vs. 76.4% for mild (P < 0.001), and 87.2% vs. 81.2% for severe (P < 0.001) (Table 5).

**Table 5:** Discriminative and Diagnostic Indices of Bradford Score, Rockall Score and UGIH Score

Indices	Score types			P-value	
	Bradford score^1^	Rockall score^2^	UGIH score^3^	3 vs. 1	3 vs. 2
Discrimination: AuROC (%)					
Mild vs. rest	87.3	70.0	86.8	0.693	< 0.001
Severe vs. rest	74.6	86.1	90.5	< 0.001	< 0.001
Diagnostic					
Sensitivity (%)					
Mild vs. rest	96.8	100	95.7	0.125	< 0.001
Severe vs. rest	9.2	16.2	62.3	< 0.001	< 0.001
Specificity (%)					
Mild vs. rest	59.0	0	68.7	< 0.001	< 0.001
Severe vs. rest	99.7	94.1	92.0	< 0.001	0.029
PPV (%)					
Mild vs. rest	88.4	76.4	90.8	0.037	< 0.001
Severe vs. rest	84.0	34.9	60.2	< 0.001	< 0.001
NPV (%)					
Mild vs. rest	85.2	-	83.2	0.146	-
Severe vs. rest	85.0	85.3	92.7	< 0.001	< 0.001
Correctly classified (%)					
Mild vs. rest	87.9	76.4	89.3	0.243	< 0.001
Severe vs. rest	85.0	81.2	87.2	0.092	< 0.001

**Figure 1:**
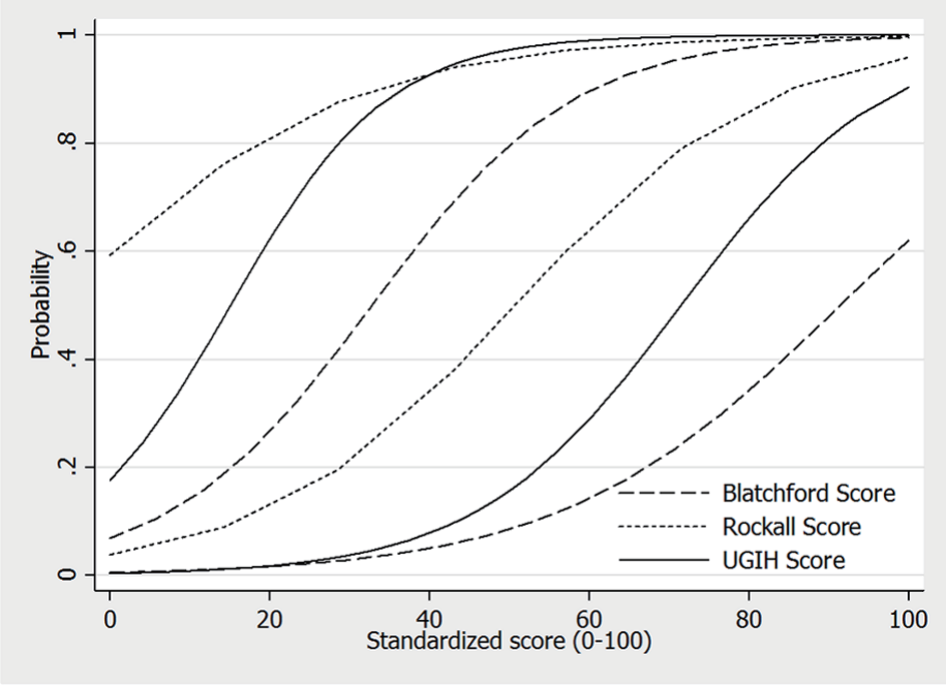
Discrimination of upper gastrointestinal hemorrhage patients into mild, moderate and severe, by the Blatchford score, the pre-endoscopic Rockall score, and the local UGIH score. Original scores were transformed into the same scaled standardized score from 0 to 100.

When exploring into the discrimination curves, the Blatchford score classified more patients into the mild categories, and less into the severe categories than the local UGIH score. In contrast, the pre-endoscopic Rockall score classified less patients into the mild, but more into the severe that the local UGIH score (Fig. 1).

## Discussion

With the aim of predicting recurrent bleeding, the Blatchford score was developed from 1,748 patients in Glasgow, Scotland, based on clinical and laboratory data, without endoscopy, and was validated for the risks (need) of clinical intervention [15, 20]. The score ranged from 0 to 23, and higher score represented higher risks [21]. The Rockall score was developed based on both clinical and endoscopic criteria, from 4,185 patients from 79 hospitals in the UK, focusing the prediction for re-bleeding and mortality. The score ranged from 0 to 7 in the clinical Rockall score, and 0 - 11 in the complete Rockall score [21]. It was recommended that low clinical Rockall score could be used as a guideline to discharge the patients safely and to appoint as OPD cases for elective endoscopy, in order to reasonably allocate resources [22].

In the original report concerning the Blatchford score [15], score 0 would signify low risks for adverse clinical outcomes, with 96% sensitivity and 32% specificity [15]. However, an external validation yielded different results with 100% sensitivity and 3.4% specificity [23]. When the cutoff value was shifted to 2, 87% of the patients with low risks were set for emergent endoscopy, reflecting an over-diagnosis [23]. On validation, the clinical Rockall score at 0 observed no adverse outcomes, at scores 1 - 3, although there were no adverse outcomes observed, blood transfusion was observed in 29%, while 21% re-bleeding, 5% surgery and 10% death were observed in score > 3 [22]. For the Rockall score, an external validation yielded poorer calibration fit in the prediction for re-bleeding than mortality. When concerning discrimination, an AuROC was lower for prediction of re-bleeding, but higher for mortality [24].

Some studies reported better performances of the Blatchford score over the pre-endoscopic Rockall score in predicting need for endoscopy [25], but with low specificity (6.3%) [25]. In Thailand, researchers claimed that the Blatchford score predicted high risk for re-bleeding and mortality better than the clinical Rockall score [26], but the score boundaries between low risks and high risks were narrow in both scoring systems. Using 0 score for low risks and > 0 for high risks classified the majority of patients as “high risk” [16] when in fact most of UGIH cases do not require endoscopic treatment, surgery, blood transfusion, and re-bleeding or death were small [5, 27, 28]. More resources would therefore be consumed unnecessarily under those circumstances [16].

Among many publications on clinical prediction rules for risk stratification of UGIH [5, 14, 15, 29-32], no single scores were widely adopted to be used in routine clinical practice [33]. This might be explained by insufficient validation [14, 15, 31, 32], low clinical applicability [5, 29, 30], or complexities [5, 8, 15, 32]. To refine UGIH classification to make it more flexible, and due to the fact that emergent endoscopy may not be readily available for every patient in all hospitals, as in Thailand or in other countries, classifying UGIH patients into three categories should enhance the practicability to selectively set for endoscopy within 24 h, especially for patients with risk risks [16].

We agreed that the Blatchford score may be suitable in case of need for intervention (blood transfusion, endoscopic hemostasis, or surgery) [1-15, 20-26], but in classifying patients into three urgency levels to set priority for endoscopy, the Blatchford may not be suitable, as some moderately severe patients would be classified as mild (under-estimate), and some severe patients would be classified as moderate (under-estimate). We also agreed that the pre-endoscopic Rockall score [5] may be suitable in the prediction for poor clinical outcomes (re-bleeding or death), but it may not be suitable for classifying UGIH severity, as some mildly severe patients would be classified as moderate (over-estimate), and some moderately severe patients would be classified as severe (over-estimate).

Adoption of a score developed for one condition to be used in another condition may be inappropriate. Choosing the cutoff point not previously mentioned in the original scoring system may cause clinically meaning misclassifications. In this report, we used AuROC to avoid an arbitrary cutoff point, and analyze sensitivity and specificity from a logistic function with the same probability of 0.5 in all three systems, and found that both AuROC and % correctly classified were in similar directions, indicating that the statistical analysis was unlikely to be biased.

The findings in this report led to the conclusion that clinical score development should be conducted in the context of clinical objectives. Any cross-contextual uses of clinical score are not likely to be successful and could lead to invalid results. It should be emphasized that the score developed in one own settings may be more suitable than adopting scores developed from another settings with different population mix.

### Conclusions

Triaging UGIH patients into three severity levels in order to decide or set for endoscopy should apply the scoring system specifically developed for that purpose. Adopting other scores developed for other purposes may result in under- and/or over-estimations. The present report clearly demonstrated that the local UGIH score classified patients into three severity levels to help indicate endoscopy more efficiently than the other two existing scoring systems (the Blatchford score and the pre-endoscopic Rockall score) which were developed for different purposes.

## Abbreviations

UGIH: upper gastrointestinal hemorrhage
AuROC: area under receiver operating characteristic curve
IPD: in-patient department
OPD: out-patient department
